# Supplementary material for: Competitive co-diffusion as a route to enhanced step coverage in chemical vapor deposition
Source: Nat Commun. 2024 Dec 11;15:10667. doi: 10.1038/s41467-024-55007-1 (PMC11634887; doi:10.1038/s41467-024-55007-1)
Supplement: Supplementary file 1 — Supplementary Information [file 41467_2024_55007_MOESM1_ESM.pdf]

Supplementary information:

Competitive co-diffusion as a route to enhanced step coverage in chemical vapor deposition

Arun Haridas Choolakkal, Pentti Niiranen, Samira Dorri, Jens Birch and Henrik Pedersen*

Department of Physics, Chemistry and Biology, Linköping University, SE-581 83 Linköping, Sweden.

* E-mail: henrik.pedersen@liu.se

Supplementary note 1:

Lower Xe flow

In our study, the step coverage observed in a 10:1 aspect ratio feature increased from 0.71 to 0.97 with the addition of 100 sccm co-flow of Xe. Interestingly, when using a 50 sccm Xe co-flow, the step coverage aligned with the observation, resulting in a value of 0.87 as presented in Suppl. Fig. 1. These observations highlight the impact of Xe flow on step coverage in the given aspect ratio.

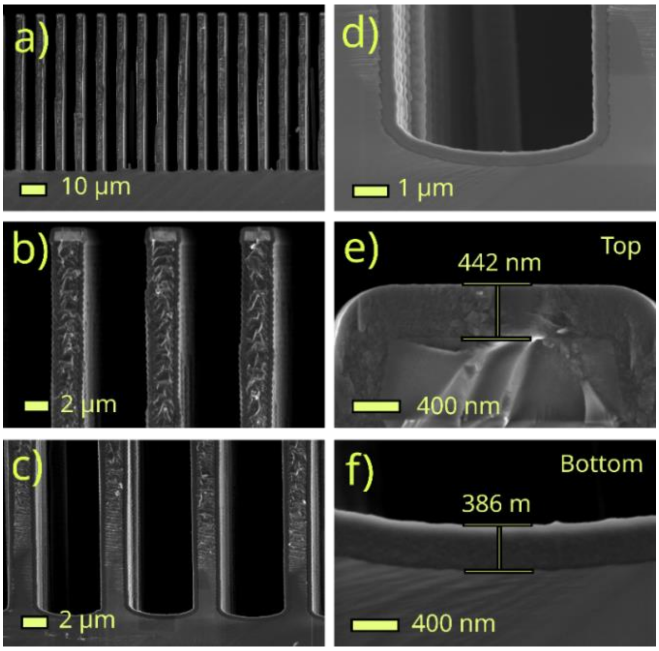

Supplementary Fig. 1 | Electron micrography of film deposited with lower Xe flow. Cross-sectional SEM micrograph obtained for the boron carbide thin film deposited in 10:1 aspect-ratio micro trench structure with 50 sccm co-flow of Xe. a) overview of the trenches, b) close-up of top of the trenches, c) bottom of the trenches, zoom in of the trench bottom, film thickness measurements at the top e), and bottom f).

Supplementary note 2:

Material characterization

The chemical bonding in the films from XPS core level spectra shows that the B 1*s* can be fitted by two distinct chemical states with energy peaks at 188.2 eV and 189 eV (Suppl. Fig. 2a). The peaks at 188.2 eV and 189 eV are attributed to B–B and B–C bonds respectively.[1,2] The C 1*s* spectra are fitted by three chemical environments with peaks centered at 282.5 eV[3], assigned to *sp*-hybridized[4] carbon bond in the C–B–C chain, 283.5[3] eV assigned to C–B bonds where sp^2-hybridized[4] C atoms bonded to the equatorial B atoms in the icosahedra and 284.5 eV[1] to sp^2 hybridized C–C bonds from the amorphous carbon phase (Suppl. Fig. 2b). The peak positions in XPS are not affected by the addition of Xe gas. The UPS measurements show a work function of about 4.2 eV which is not affected by the addition of Xe (Suppl. Fig. 2c). The valance band scan shows a wide spectrum without any sharp fermi edge (Suppl. Fig. 2d) suggesting that the material is not a good electrical conductor. These results shows that the competitive co-deposition process with Xe does not alter the chemical and electronic nature of the boron carbide film.

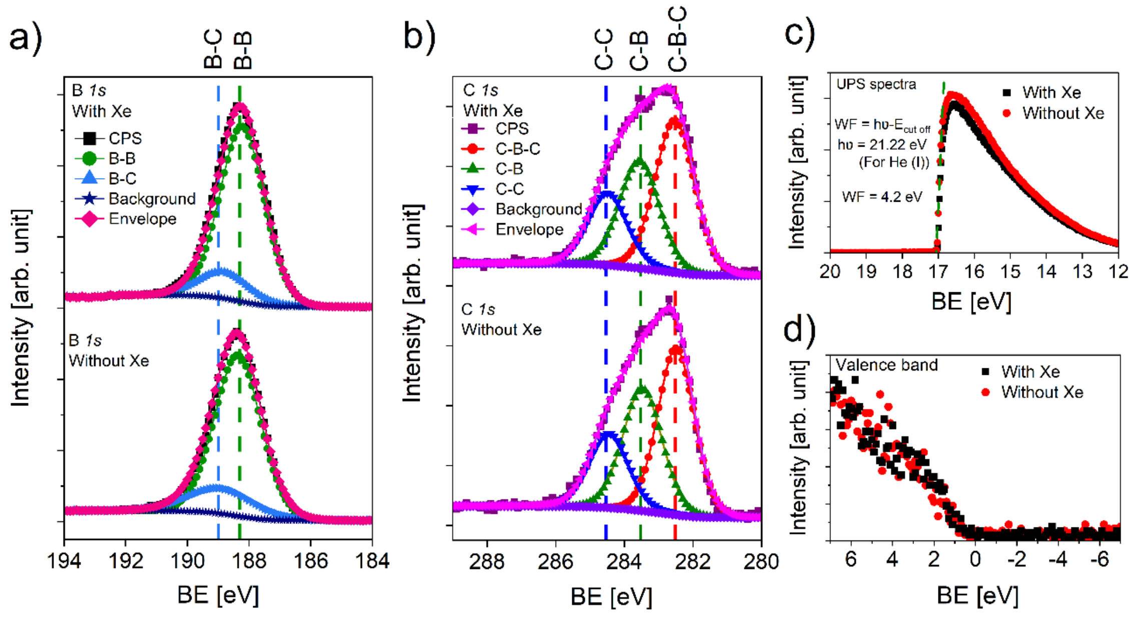

Supplementary Fig. 2 | Material characterization by XPS and UPS. Spectra obtained for the samples deposited both in Xe containing ambient and Xe free ambient at 550 °C. a) B 1*s* core level spectra, b) C 1*s* core level spectra, c) work function measurement obtained by operating the ultraviolet source in the XPS chamber and d) the valence band scans of the samples. Source data are provided as a Source Data file.

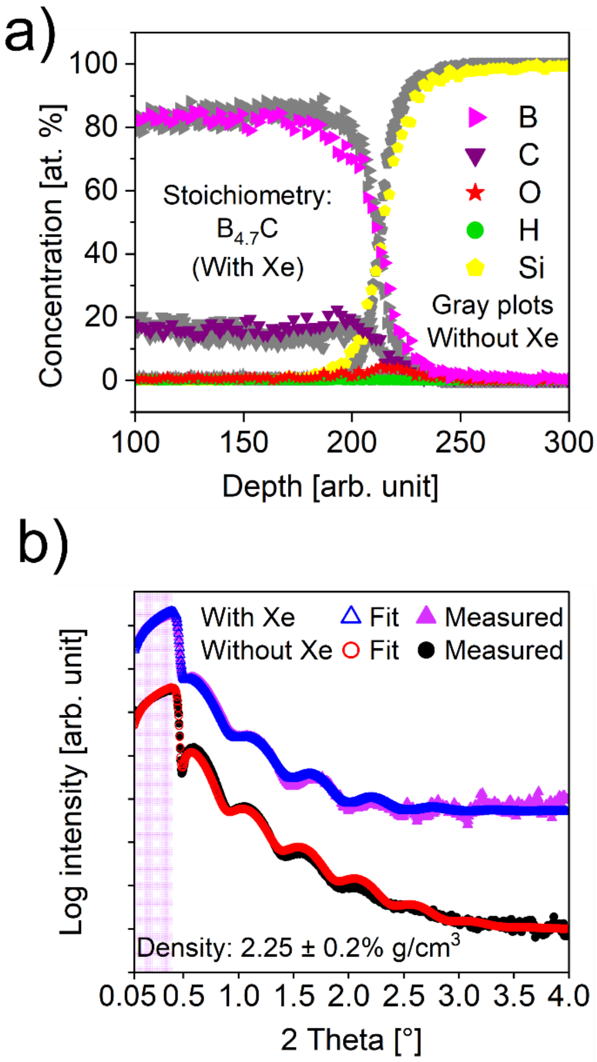

Supplementary Fig. 3 | Material characterization by ToF ERDA. a) Depth profile of elemental composition obtained by time-of-flight elastic recoil detection analysis (ToF ERDA) measurements for the sample deposited with Xe. The gray curve, composition obtained for the samples deposited without Xe, provided for a comparison. b) X-ray reflectivity (XRR) curves fitted for density and thickness of the 16 nm thick films deposted in Xe containing ambient and Xe free ambient show similar density values. Source data are provided as a Source Data file.

Supplementary note 3:

Diffusion modeling

By ignoring any surface reactions, the competitive diffusion behavior of TEB and Xe in a gas mixture with H_2 ambient can be characterized by the ratio of their diffusion coefficients and their relative concentrations in the gas phase. TEB and Xe diffuse towards the trench bottom with two different diffusion rates, the flux ratio between Xe and TEB varies as a function of trench depth and time. The TEB concentration over a 100 µm deep and 6 µm wide trench structure was approximated by solving Fick's second law using a MATLAB program. The diffusion rate of Xe in the H_2 ambient (D_{XeH}) was calculated using Chapman and Enskog's equation: [5,6]

$$D_{XeH} = \frac{3}{16} \frac{(4\pi kT/M_{XeH})^{1/2}}{n\pi\sigma^2{}_{XeH}\Omega_D} f_D \quad (1)$$

Where k = Boltzmann's constant

T = temperature in absolute scale

n = number density of molecules in the mixture

M_{Xe} and M_H = molecular mass of Xe and H

$M_{XeH} = 2[(1/M_{Xe}) + (1/M_H)]^{-1}$

σ_{XeH} = characteristic length of the intermolecular force law

Ω_D = diffusion collision integral given by kT/ε_{XeH}.

f_D = correction term (chosen as unity)

The Lennard-Jones potentials (σ and ε) were taken from Suppl. Ref. 5, and the values were σ (Xe) = 4.047 Å, ε (Xe) = 231 K, σ (H_2) = 2.827 Å and ε (H_2) 59.7 K. For collision between two different species, equations 2 and 3 were used:[5]

$$\sigma_{ij} = (\sigma_i + \sigma_j)/2 \quad (2)$$

$$\varepsilon_{ij} = (\varepsilon_i \, \varepsilon_J)^{1/2} \quad (3)$$

The effective diffusion rates in high aspect ratio structure were then estimated by multiplying the derived diffusion rates with the average open space[7] between the trench structures. The diffusion rate for TEB was then approximated using Grahams law. Further, these values were used to solve the Fick's equation (4),[8,9,10] to estimate the concentration gradient in one

dimensional scenario. If, φ is the concentration in one dimension, t is time in seconds, D is the diffusion coefficient, and x is the trench depth, the change in concentration with depth and time is given by:[8,11]

$$\frac{\partial \varphi}{\partial t} = D\frac{\partial^2 \varphi}{\partial x^2} \tag{4}$$

All other process conditions such as partial pressure, substrate temperature, and the total reactor pressure were taken as the same as the actual process. We assume a uniform temperature over the microscale features and thus a constant reaction probability around it. Meaning that the deposition rate depends only on partial pressures of the film forming species. Both TEB and Xe fluxes were calculated as a function of trench depth and time with the assumption that no reactions were present and are depicted in Suppl. Fig. 4. Given TEB's behavior, we make the assumption that its sticking probability is significantly less than 1. Consequently, the precursor molecules predominantly diffuse back in the gas phase rather than rapidly reacting at the surface. Also, we maintained two orders of magnitude higher flow rate for hydrogen gas compared to Xe and TEB to mitigate the hydrogen partial pressure's impact on the reaction rates across the trench depth. Furthermore, the Knudsen number (Kn), which compares the molecular mean free path (λ) length to a representative physical length scale, becomes crucial.

The λ and the corresponding Kn values calculated using equations 5 and 6 show an empirical flow regime classification as transitional flow with Kn about 1 to 1.1. This regime finds a balance between effective mass transport and desired gas phase collisions. Furthermore, Fick's equation remains adaptable for transitional flow conditions.[12]

$$\lambda = \frac{RT}{\sqrt{2}\,\pi d^2 N_A P} \tag{5}$$

Where, R is gas constant, N_A is Avogadro's number, pressure (P) = 5 kPa, Temperature (T) = 823 K (550 °C), collision cross section (d) = 2.89 x 10^{-10}.

$$Kn = \frac{\lambda}{L} = 1 \text{ to } 1.1 \tag{6}$$

Where, λ = 6.612 µm and the representative physical length scale (L) = 6 to 6.5 µm

It can be noted from Suppl. Fig. 4 that when TEB and Xe gases were simultaneously introduced, the flux ratio varies from 1.23 % to 1.37 % over 100 µm trench depth in 0.4 µs. However, this

concentration gradient decreases as a function of time and eventually reaches an equilibrium. The TEB flux drop towards the trench bottom is partially compensated by the higher TEB to Xe concentration towards the bottom surfaces. Since TEB is being consumed to form solid film during the deposition process, there will always be a pressure drop towards the bottom of the trench which needs to be leveled by further diffusion of the reactants through the capillary like trench structures. Although the intense competitive behavior diminishes due to the saturation of Xe gas partial pressure over the trench depth, the film formation keeps the diffusive mass transport alive.

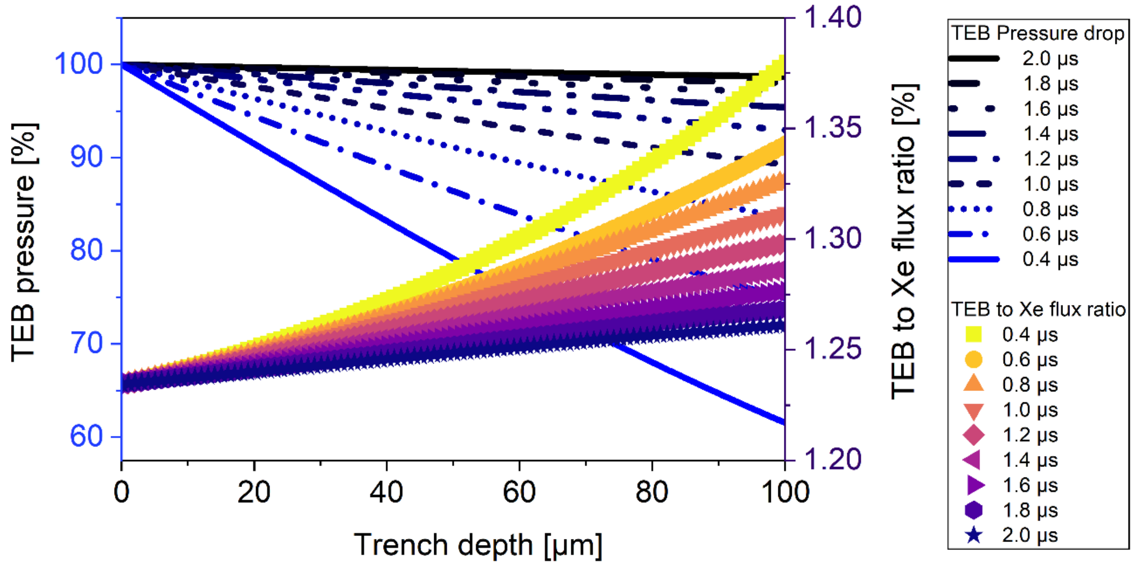

Supplementary Fig. 4 | Partial pressures in the trenches. TEB partial pressure in percentage (left Y-axis) as a function of trench depth for a 6 μm wide trench for various time is plotted. The partial pressure in the top surface is considered as 100 %. The flux ratio of TEB/Xe (right Y-axis) as a function of trench depth for various time is plotted. It was assumed that no surface reactions were present. We considered 1 sccm (1.18 Pa) flow of TEB and 100 sccm (112 Pa) flow of Xe at 550 °C in H_2 ambient gives 5 kPa total pressure. Source data are provided as a Source Data file.

It can be further noted from Suppl. Fig. 5 that, in addition to the enhancement in the local relative concentration of TEB towards the trench bottom, TEB flux towards the trench bottom increases with Xe addition. This means, that at the bottom surfaces, both absolute and relative concentration of the TEB molecule increases with Xe co-flow. While trench deposition takes several minutes, the shown effect is short-lasting. On the other hand, these calculations, limited to a feasibility test of Xe as a co-diffusion pair for TEB, assume no surface reactions. However,

in reality, steady-state mass transport due to film deposition and lighter species generation occurs.

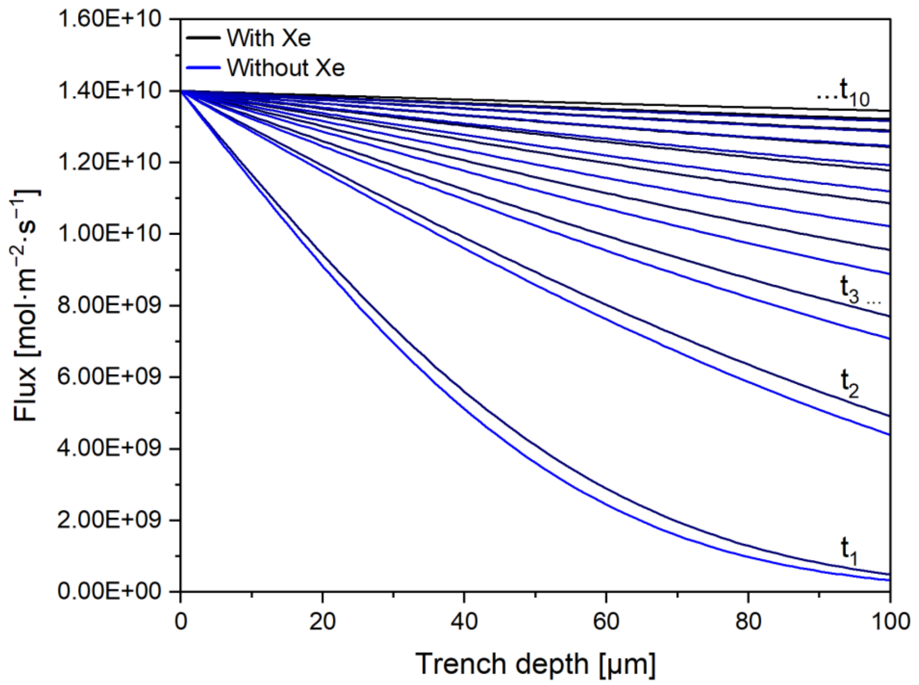

Supplementary Fig. 5 | Flux of molecules in the trenches. TEB flux with and without Xe as a function of trench depth for a 6 μm wide and 100 μm deep trench for various time (ti). It was assumed that no surface reactions were present. We considered 1 sccm (1.18 Pa) flow of TEB and 100 sccm (112 Pa) flow of Xe at 550 °C in H_2 ambient gives 5 kPa total pressure. Source data are provided as a Source Data file.

This led us to try experiments with a pulsed Xe flow. If the partial pressure of Xe is allowed to drop during the time between the Xe pulse, a pulse of Xe will increase it to enhance the competitiveness in the co-diffusion and keep the TEB to Xe flux ratio at the bottom surfaces on the higher side. We tested this potential evolution of the competitive diffusion concept and Suppl. Fig. 6 shows the cross-section SEM micrographs obtained for a film deposited with a pulsed delivery of Xe gas with 1 s Xe flow (100 sccm, corresponding to a partial pressure of 18 Pa) after every 30 s. From the cross-section SEM micrographs, we note that the obtained SC from the pulsed delivery of Xe is similar with the obtained SC from the continuous flow. However, with the pulsed delivery of Xe gas, the deposition rate has decreased from 7.2 to 2.85

nm/min. We attribute this to the dynamic pressure inside the reactor causing a periodic dilution of TEB concentration, consequently, a decrease in the incident flux of reactive species.

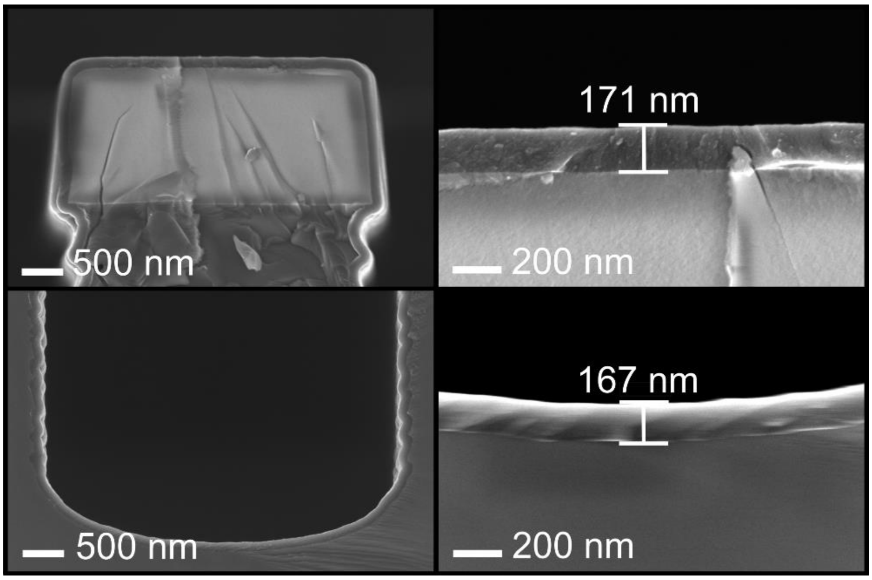

Supplementary Fig. 6 | Electron micrography of a film deposited with a pulsed Xe flow. SEM micrograph showing boron carbide film deposited in 10:1 aspect ratio feature at 550 °C substrate temperature with a pulsed supply of Xe gas.

Supplementary References

1. Imam, M. *et al.* Trimethylboron as single-source precursor for boron-carbon thin film synthesis by plasma chemical vapor deposition. *J. Phys. Chem. C* **120**, 21990–21997 (2016).

2. Imam, M. *et al.* Plasma CVD of hydrogenated boron-carbon thin films from triethylboron. *J. Chem. Phys.* **148**, 034701 (2018).

3. Imam, M. *et al.* Gas Phase Chemistry of Trimethylboron in Thermal Chemical Vapor Deposition. *J. Phys. Chem. C* **121**, 26465–26471 (2017).

4. Mondal, S. *et al.* Disorder and defects are not intrinsic to boron carbide. *Sci. Rep.* **6**, 11–16 (2016).

5. Poling, B. E., Prausnitz, J. M. & O'Connell, J. P. *The properties of gases and liquids. Solutions* (McGRAW-HILL, 2011). doi:10.1036/0070116822.

6. Acosta, R. H. *et al.* Diffusion in binary gas mixtures studied by NMR of

hyperpolarized gases and molecular dynamics simulations. *Phys. Chem. Chem. Phys.* **8**, 4182–4188 (2006).

7. Cremers, V., Geenen, F., Detavernier, C. & Dendooven, J. Monte Carlo simulations of atomic layer deposition on 3D large surface area structures: Required precursor exposure for pillar- versus hole-type structures. *J. Vac. Sci. Technol. A Vacuum, Surfaces, Film.* **35**, 01B115 (2017).

8. Muir, C. E., Lowry, B. J. & Balcom, B. J. Measuring diffusion using the differential form of Fick's law and magnetic resonance imaging. *New J. Phys.* **13**, 015005 (2011).

9. Owusu, J. P., Karalis, K., Prasianakis, N. I. & Churakov, S. V. Diffusion and Gas Flow Dynamics in Partially Saturated Smectites. *J. Phys. Chem. C* **127**, 14425–14438 (2023).

10. Fayaz-Torshizi, M. *et al.* Use of Boundary-Driven Nonequilibrium Molecular Dynamics for Determining Transport Diffusivities of Multicomponent Mixtures in Nanoporous Materials. *J. Phys. Chem. B* **126**, 1085–1100 (2022).

11. Westwater, J. W. & Drickamer, H. G. The Mathematics of Diffusion. *J. Am. Chem. Soc.* **79**, 1267–1268 (1957).

12. Yim, J., Verkama, E., Velasco, J. A., Arts, K. & Puurunen, R. L. Conformality of atomic layer deposition in microchannels: impact of process parameters on the simulated thickness profile. *Phys. Chem. Chem. Phys.* **24**, 8645–8660 (2022).
